# Nausea, vomiting and conflict in pregnancy: The adaptive significance of Growth-Differentiation Factor 15

**DOI:** 10.1093/emph/eoae008

**Published:** 2024-04-23

**Authors:** Bernard J Crespi

**Affiliations:** Department of Biological Sciences, Simon Fraser University, Burnaby, BC, Canada

**Keywords:** nausea, vomiting, pregnancy, GDF15, evolution, conflict

## Abstract

Nausea and vomiting in pregnancy (NVP) is heritable, common and aversive, and its extreme, hyperemesis gravidarum (HG), can be highly deleterious to the mother and fetus. Recent influential studies have demonstrated that HG is caused predominantly by high levels of Growth-Differentiation Factor 15 (GDF15), a hormone produced by the placenta in substantial amounts. This work has led to calls for therapeutic modulation of this hormone to reduce GDF15 levels and ameliorate HG risk. I describe three main lines of evidence relevant to the hypothesis that GDF15 production is typically adaptive for the fetus, in the context of enhanced placental invasion, reduced rates of miscarriage and preterm birth and higher birth weight. These considerations highlight the medical implications of maternal-fetal conflict, in the context of tradeoffs between aversive symptoms during gestation, rare disorders of pregnancy with major adverse effects and moderate fitness-enhancing benefits to fetuses.

## INTRODUCTION

A key tenet of evolutionary medicine is that traits that are aversive and unpleasant can also be adaptive [1]. This principle is important because medical interventions to reduce the expression of aversive yet adaptive traits can have deleterious effects on both fitness and health. For example, low mood, anxiety, fever and anemia from infection have been demonstrated to provide physiological and fitness-related benefits, yet are usually considered as detrimental and treated as such [1].

Human gestation exhibits a number of aspects that are notably adverse. Prominent among them is nausea and vomiting of pregnancy (NVP), which is reported by up to about 80% of women especially during the first trimester [2], and is highly heritable [3]. NVP reduces food intake and produces aversions to particular foods. It varies continuously in degree from mild to an extreme referred to as hyperemesis gravidarum (HG), which typically affects up to about 1–2% of pregnancies and involves intractable vomiting, weight loss and dehydration, that can become injurious to the mother and fetus [4, 5]. Treatment of NVP and HG remains challenging due to the potential effects of therapy on the first-trimester fetus, which is especially vulnerable to potential harmful impacts from systemic medications.

NVP and HG have for many years been attributed to the effects of human chorionic gonadotropin, a hormone produced by the placenta in early development that stimulates the corpus luteum to generate progesterone and thus maintain the pregnancy [5–7]. The primary evidence for this inference is a coincidence in the timing of human chorionic gonadotropin production with NVP symptoms, with both peaking at around 8–12 weeks of pregnancy.

Over the past few years, culminating in an influential paper in 2023 [8], NVP and HG have instead been shown to be caused predominantly by relatively high levels of a hormone called Growth-Differentiation Factor 15 (GDF15). GDF15, like human chorionic gonadotropin, is secreted by the placenta into maternal circulation starting in early pregnancy; higher levels are causally associated with stronger NVP and higher risk of HG, as evidenced by a suite of convergent genetic, genomic, observational and experimental studies [8]. Moreover, especially high NVP levels are conferred by relatively low pre-pregnancy GDF15, followed by relatively high GDF15 during gestation [8]. These findings have motivated proposals for novel therapies to reduce NVP and HG, based on blocking the effects of GDF15 during pregnancy, as well as by increasing its pre-pregnancy levels [8].

The purpose of this article is to describe and evaluate the hypothesis that levels of GDF15 sufficient to cause NVP are, in the majority of cases, adaptive for the developing fetus. As such, therapies based on reducing the physiological effects of this hormone, while they may reduce the risk of HG, are also expected to have some degree of deleterious impacts on most pregnancies in terms of placental development, early maintenance of pregnancy, birth timing, birth weight or some combination of these effects.

I first briefly describe relevant evolutionary theory for maternal-fetal interactions and then situate GDF15 within this context. Next, I present three main lines of evidence salient to the hypothesis that GDF15 production by the placenta is typically adaptive for the fetus. I conclude with implications for the prevention or treatment of NVP and HG.

## THEORY AND PHYSIOLOGY OF MATERNAL-FETAL CONFLICT

About 30 years ago, Haig [9] first described the evolutionary logic of maternal-fetal conflicts, which follows from pioneering work by Trivers [10]. Maternal-fetal conflict is predicated on the fact that the mother and fetus and genetically related by only one-half, such that their interactions involve a mix of cooperation (over the success of the pregnancy, given its viability) and conflict (over the extent of resource provision to the fetus, such that a fetus is selected to solicit, and if possible acquire, more resource than the mother is selected to provide to it) [9]. Such conflict is enacted biochemically, usually through the secretion of hormones by the fetal-placental unit into the maternal bloodstream, with maternal physiological responses that are selected to mitigate fetal hormonal effects that exceed her optimal investment in a given fetus [11]. In particular, fetuses are selected for effects that lead to effective placental invasion, maintenance of the pregnancy, higher (but not too high) maternal glucose levels and blood pressure leading to higher birth weight, and birth timing at term [9, 11, 12]. Mildly deleterious or aversive effects on the mother are expected in such systems, provided that they increase the inclusive fitness of the fetus overall.

The physiological effects of well-studied placental hormones, including, for example, human chorionic gonadotropin, human placental lactogen and placental growth hormone, fit with maternal-fetal conflict theory, as do the causes of major disorders of pregnancy, including gestation diabetes, intrauterine growth restriction and pre-eclampsia [9, 11–14]. As such, these disorders appear to represent, in large part, maladaptive extremes or dysregulation of conflict-related adaptations of the fetus or mother, and they can be fully understood only within this conceptual framework.

## THE ADAPTIVE SIGNIFICANCE OF GDF15

How do the functions and effects of GDF15 fit within the paradigm of maternal-fetal conflict, and the adaptive significance of placental hormones? GDF15 is normally produced at high levels only from the placenta and released into maternal circulation [15]. Its receptor, called GDNF family receptor α-like (GFRAL), is localized to specific regions in the hypothalamus and hindbrain that control appetite and other physiological mechanisms [16, 17]. GDF15 regulates a variety of functions, including food intake (via appetite and nausea induction), expenditure of energy, and bodily responses to stresses including infection and injury [15, 18]. These roles are mediated by various mechanisms, including increases in catabolism relative to anabolism, as indicated, in part, by increases in serum glucose and triglycerides in association with increased GDF15 levels among healthy, non-pregnant subjects [15, 19, 20].

A first line of evidence concerning the adaptive significance of GDF15 in pregnancy is based on its recently documented role in causing NVP and, at the extreme, HG. Although NVP has adverse psychological impacts in the contexts of anxiety, depression and quality of life (e.g. [21, 22]), considerable evidence indicates that it is associated with positive effects on birth outcomes with regard to reduced incidence of miscarriage [23–26], reduced rate of preterm birth [27–30], and higher birth weight (reviews in [29, 31]). These reported positive effects are by no means detected by all relevant studies but combined with the fact that NVP is found in most pregnancies (and as such is much less a ‘sickness’ than a norm), they indicate that NVP, and hence its primary cause GDF15, commonly exhibits adaptive consequences for the fetus and mother. By contrast, HG clearly represents a rare, maladaptive extreme of NVP, just as gestational diabetes represents a harmful extreme of elevated maternal serum glucose, and pre-eclampsia represents a deleterious extreme of elevated maternal blood pressure.

Adaptive effects of NVP on birth outcomes have usually been attributed to avoidance and expulsion of noxious or teratogenic foods in early pregnancy, when embryos are relatively vulnerable to developmental disruptions (e.g. [25]). This hypothesis is consistent with some patterns of food aversions and preferences in pregnancy, such as avoidance of meat, and with data showing that women with NVP have reduced rates of some birth defects [32]. However, nausea and vomiting due to other causes, such as cancer treatments, induce similar food aversions [33], indicating that such effects are not specific to pregnancy. Moreover, the association of lower NVP with higher rates of birth defects may be due to reduced embryo viability causing both outcomes, rather than to a causal chain from NVP, to food aversions, to birth defects. Given that there is no experimental or observational evidence directly linking reduced NVP with the causes of birth defects, this particular hypothesis requires additional testing. Similarly, although some infections (e.g. with *Listeria* or *Toxoplasma*) from meat or dairy are known to cause miscarriages [34], their relationship to NVP remains unclear. Thus, although avoidance of toxicity and infection via NVP may provide benefits to mothers and fetuses, this hypothesis is as yet unsubstantiated.

What, if not reduced infection and toxicity (or in combination with such effects), might be the causes of reduced miscarriage and higher birth weights found among women with NVP? A second line of evidence for the adaptive function or functions of GFD15 is its recently-demonstrated role in the stimulation of early placental development and invasiveness via the JAG-1, TGF-β and Smad1/5 pathways [35, 36] and data demonstrating that reduced levels of this hormone are associated with higher rates of miscarriage [35, 37–39]. Specifically, reduced levels of GFD15 have been reported in placental and serum samples of women with unexplained recurrent pregnancy loss, compared to controls, and in mice, reduced placental and serum GDF15 have been associated with increased embryo resorption (the murine equivalent of miscarriage), which could be reversed by GDF15 administration [35, 36]. These effects, which are mediated by placental invasiveness, provide support for the longstanding hypothesis, set forth by Rachel Huxley [40], that the adaptive significance of NVP derives from reduced early pregnancy food intake leading to increased maternal catabolism (and lowered anabolism), resulting in enhancement of early placental development [31, 40] that can reduce rates of pregnancy loss. Given the high rates of early clinical and pre-clinical miscarriage found in human pregnancies [9], selective advantages of GDF15 production in this regard should be especially strong. Relatively low food intake in early pregnancy, or reduced early pregnancy intake of specific foods, have also been associated with better placental development and reduced fetal loss in other contexts, suggesting mechanistic connections between NVP, which reduces food intake, and benefits to the fetus [31, 41, 42]. Such effects are further supported by the evidence that GDF15 reduces appetite and food intake [43, 44], and promotes catabolic activity [15], including data showing associations of higher serum GDF15 with reduced body weight or body mass index among women [45, 46] and mice [47, 48].

Fetal growth is mediated most notably by maternal serum glucose, which is directly accessible to the placenta in species like humans and mice that have hemochorial placentation [9]. A third line of evidence concerning the adaptive significance of GDF15 in pregnancy is data relating it to maternal levels of glucose. Thus, higher GDF15 has been linked with increased maternal serum glucose in pregnancy [49], and levels of this hormone are higher among women who develop gestational diabetes [50–52]. Among typical pregnant women, elevated maternal glucose leads to increased birth weights [53], with gestational diabetes in the mother and fetal macrosomia resulting at the extreme. By this mechanism, the benefits of GDF15 derive from enhanced maternal catabolic activity (relative to anabolism), which increases levels of glucose (and possibly other nutrients such as triglycerides) available to the fetus [31, 40]. This hypothesis is also supported by evidence for positive associations of maternal or infant serum GDF15 with birth weight [50, 54] although further such data are needed especially from non-clinical populations.

Maternal responses to placental GDF15 production are, in turn, suggested by the finding that increases in this hormone lead to enhanced maternal β-cell proliferation and insulin production (that are under maternal control) [44, 55, 56], which have the effect of reducing maternal glucose—thus exemplifying the expected tug-of-war conflict over maternal provisioning [9, 11, 12, 57]. In mice, GDF15 has also recently been shown to act as a ‘rheostat’ to protect against hypoglycemia, through the induction of gluconeogenesis [58]. This particular function, if demonstrated in human pregnancy, would further support a role for this hormone in fetal regulation of maternal glucose. These effects and correlates of GDF15 are notably convergent with those of other placentally expressed hormones, including human chorionic gonadotropin, human placental lactogen and human placental growth hormone, in that higher expression of these hormones have been associated with increases in correlates of offspring fitness [9, 11, 12].

**Figure 1.**
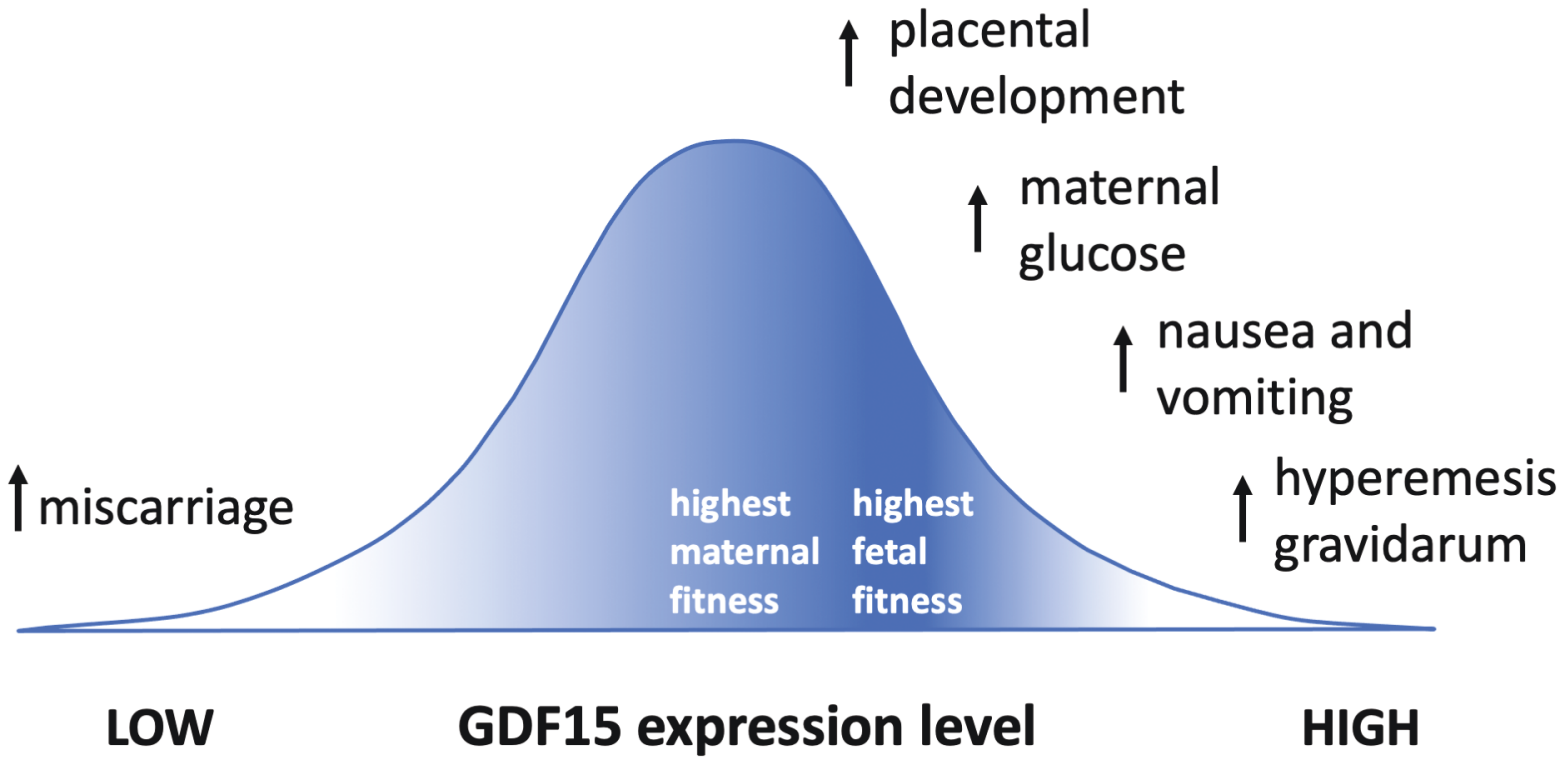
Effects of different levels of GDF15 expression on mechanisms and causes of fetal and maternal fitness. Darker shading represents higher fetal inclusive fitness. By this hypothesis, the optimum level of GDF15 is higher for the fetus than for the mother, due to its positive effects on fetal growth.

**Figure 2.**
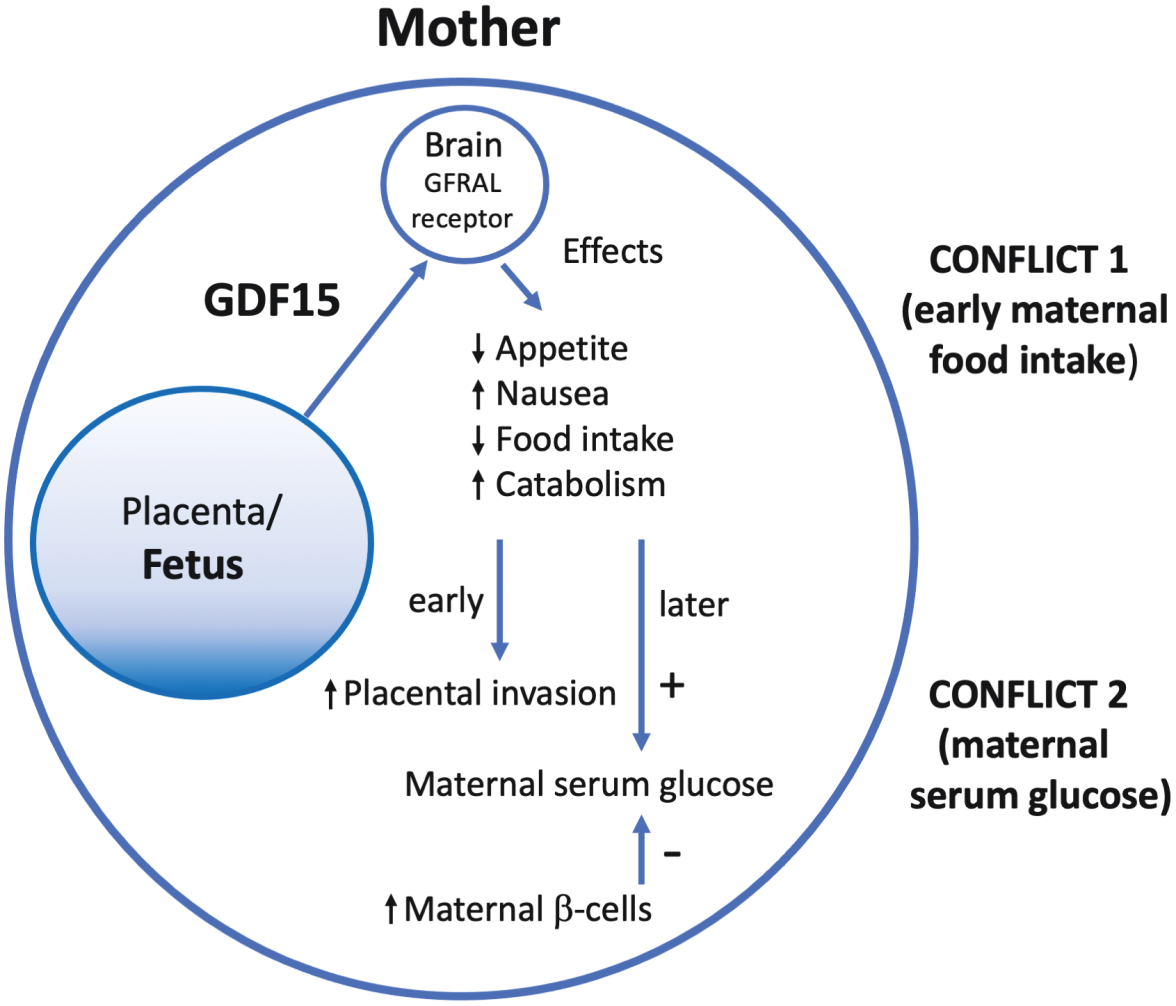
Proposed mechanisms whereby increased GDF15 expression can confer higher fitness on fetuses and whereby mothers can counter fetal fitness-enhancing effects via reducing maternal levels of serum glucose.

Hypotheses for the adaptive significance of GDF15, in the contexts of NVP and maternal-fetal conflicts, focus on the benefits and costs of its levels, from too low, to optimal for the mother, to optimal for the fetus, to too high (Fig. 1). The demonstrated physiological roles of GDF15 in (i) causing NVP (which has been associated with better pregnancy outcomes), (ii) promoting placental invasion (which reduces rates of early miscarriage) and (iii) leading to increased maternal serum glucose (which enhances fetal nutrition) thus support the hypothesis that high expression of this hormone from the placenta represents a manifestation of maternal-fetal conflict over levels of resource provision. This conflict is expressed in two main contexts. First, maternal food intake during early pregnancy is displaced from the maternal optimum by the appetite-suppressing and nausea-inducing effects of GDF15, which apparently leads to increased maternal catabolism (relative to anabolism) that enhances placental invasion [40] (Fig. 2). The evolution of reduced maternal responsiveness to GDF15, through GFRAL receptor stimulation in the hypothalamus and hindbrain, is presumably limited by the physiological importance of GDF15 in other contexts, including stress, disease and injury [15]. Second, GDF15 shows evidence of mediating conflict over maternal serum glucose levels later in pregnancy, via induction of maternal glucose production, an effect that is countered by maternal increases in the secretion of insulin [44, 56] (Fig. 2). The outcome of this latter conflict cannot be predicted *a priori*, since it is expected to depend upon the strength of selection on both parties, the phenotypes available to them, and any constraints on optimization due to pleiotropy. However, given tug-of-war dynamics, one possible outcome would be a dynamic equilibrium without notable benefits to either mother or fetus in many or most cases, though with conflict-related physiological costs to both. Indeed, a unique signature of such genomic-conflict systems (in addition to high rates of hormone production by the placenta) is that mutational or experimental knockout of the conflict can result in little to no phenotypic effects at all (because both parties ‘drop the rope’ in the dynamic tug of war), as described by Haig [9] for human placental lactogen, and as found for knockouts involving GDF15 [8,15].

A useful parallel, as regards understanding the GDF15 system and its evolution, comes from the only other context where this hormone is expressed at extremely high levels: in cancer [15, 18, 59]. In a microarray study of 150 human carcinomas from 10 anatomical sites, GDF15 showed the highest level of tumor-associated expression of any protein [60]. The functional significance of such high expression is apparently related to the induction by GDF15 of cachexia, the rapid, extreme reduction in lean body mass, due to loss of appetite and increased catabolism, that represents a primary cause of death from cancer [18]. These findings suggest a coincidence in GDF15 function between high expression by cancers and high expression by the placenta: in both situations, the effect is to promote the release of nutrients into the general circulation, where they can be taken up for growth of the tumor or the fetal-placental unit. As such, high GDF15 expression in cancers apparently represents an example of ‘co-option’ by cancers of mechanisms that evolved in the context of invasive, hemochorial placentation, a pattern seen for many other molecules and pathways as well [61, 62]. Promotion of catabolism indeed appears to represent a primary function and correlate of GDF15 in many contexts, including infection, injury, frailty and aging [15, 63, 64].

## CONCLUSIONS AND IMPLICATIONS

Higher levels of GDF15 have been associated with a suite of apparent benefits to fetuses, including enhanced placental development, reduced rates of miscarriage and increased availability of glucose. In turn, NVP, which is now known to be caused predominantly by GDF15, also shows notable evidence of being adaptive, except in the extreme with the development of HG. Therapies for reducing the risk of HG, by lowering levels of GDF15 through ‘desensitizing’ the receptor prior to pregnancy, or antagonizing its action during pregnancy [8], can thus usefully be evaluated in the context of tradeoffs between (i) the common, aversive effects of NVP on the mother and the rare but severe medical impacts of HG on the mother and fetus and (ii) the benefits of higher GDF15, and non-severe NVP, to the fetus. Whereas the former costs are highly overt, most of the latter benefits are more subtle, and unlikely to be noticed unless explicitly addressed through targetted collection of data.

The primary medical import of these evolutionary considerations is 3-fold. First, reducing levels of GDF15 pharmacologically, by any means, is predicted to increase risks of poor pregnancy outcomes (e.g. miscarriage or lower birth weight), except when its levels would otherwise have been sufficiently high to cause HG. As such, a conservative strategy would be to restrict GDF15-based treatments to situations where HG was evident but in its early stages, rather than use such therapies to prevent or reduce NVP itself. With this approach, pre-pregnancy treatment, aimed at reducing the risk of HG by desensitizing the GDF15 receptor [8], would thus not be recommended because it would be expected, in some proportion of cases, to reduce GDF15 levels in situations when HG would not have occurred anyway. Second, prospective studies are needed on the magnitudes of the effects of variation in GDF15, before and during pregnancy, on placental development, rates of miscarriage and preterm birth, maternal glucose levels, and birth weights, to evaluate the risks associated with human manipulation of this evolved system. Finally, future studies on GDF15 and its effects need to take account of conflict-related dynamics, which often involve strong selection, evolutionary escalation, and increased scope for dysregulation causing disease [65, 66]. Such considerations are especially important given the recent intense interest in this protein’s roles in body weight regulation [67], cachexia [18] and cancer [59], as well as in pregnancy.
